# 83 Concomitance of primary systemic lupus erythematosus and Wilson’s disease in a male child: a case report

**DOI:** 10.1093/rheumatology/keac496.079

**Published:** 2022-10-07

**Authors:** Djohra Hadef, Samy Slimani, Fatiha Lahouel, Bouthaina Benlahcen, Nadira Bouchair

**Affiliations:** 1 Department of Pediatrics, University Hospital Center of Batna, Faculty of Medicine, Batna 2 University; 2 Atlas Clinic of Rheumatology; 3 Department of Nephrology, University Hospital Center of Batna, Faculty of Medicine, Batna 2 University; 4 Private Practitioner, Batna; 5 Faculty of Medicine, Badji Mokhtar Annaba University, Annaba, Algeria

## Abstract

**Background:**

Wilson’s disease (WD) is a rare, recessively inherited disorder of copper metabolism with its accumulation in multiple organs particularly in the liver and brain. Systematic lupus erythematosus (SLE) is an autoimmune disease like Wilson’s disease, involves multiple organs and systems. The combination of Wilson's disease and systemic lupus erythematosus (SLE) is not usual apart from iatrogenism.

**Objectives:**

we present a pediatric case of concurrent Wilson’s disease and primary SLE not induced by penicillamine. After extensive research in the literature, this is the only male case described so far. The other seven cases reported are female.

**Case presentation:**

A previously well 12-year-old boy was admitted to University Hospital Center of Batna (Algeria) with acute haemolysis (pallor, subictereus, and red urine). There was no consanguinity, or family history of note. Physical examination revealed normal development and growth, no fever, no lymphadenopathy, no hepatomegaly and splenomegaly. A complete blood count (CBC) revealed normochromic normocytic anaemia, thrombocytopenia, and leucopenia. His blood biochemistry showed hepatic cytolysis, and hepato-cellular insufficiency. Wilson’s disease was suspected because of the combination of the impaired liver function, hemolytic anaemia, and normal alkaline phosphatase levels. Serum ceruloplasmin and copper levels were decreased, while urinary copper was elevated confirming the diagnosis of Wilson’s disease. There was no neurological or ophthalmologic involvement. Family investigation revealed Wilson's disease with cirrhosis in a 9-year-old brother.

The onset of nephrotic syndrome and the presence of inflammatory syndrome cannot be explained by Wilson’s disease. The kidney biopsy histopathology revealed nephritis lupus class II (WHO classification). Subsequent serum analysis also revealed positive native anti-DNA and anti-PCNA antibodies verified on a second sample. Based on all the findings, the final diagnosis for this patient was Wilson’s disease combined with SLE. We started therapy with bolus of corticosteroids and Cyclophosphamide, relayed by Mycophenolate Moftil and hydroxychloroquine Cooper chelation has also been initiated.

Improvement in renal and even hepatic damage was noted. Unfortunately, after two years, the patient presented abnormal movements with dysarthria. Brain MRI showed abnormal signals of the basal ganglia consistent with neurological damage in Wilson's disease.

**Discussion:**

concomitant SLE and WD without penicillamine treatment is rare (7 cases reported in the literature with 3 children). To our knowledge, this is the first report of an association between Wilson's disease and SLE in male case. For our patient, SLE and Wilson’s disease were diagnosed simultaneously as 4 described cases.

Wilson’s disease was first suspected due to unexplained impaired liver function with hemolytic anaemia. Copper Tests confirming the diagnosis. At that time, there was no neurological or ophthalmological impairment.

For this patient, the worsening of the hematological involvement (pancytopenia) in an inflammatory context, with installation of a nephrotic syndrome cannot be explained by Wilson's disease. SLE was evoked despite the fact that it was a child and male. The PBR as well as the immunological workup were in favor of SLE disease.

Treatment of SLE improved symptoms but later chelation could not prevent the usual neurologic complication of Wilson’s disease at this age. The neurological involvement appeared at the age of 14 as described in the literature with the common sign dysathria, followed by the installation of abnormal movements due to the impairment of the basal ganglia objectived by MRI.

**Conclusion:**

Wilson’s disease and SLE not induced by penicillamine can co-exist. As there is no pathophysiological explanation, it’s probably a simple fortuitous association.

